# Evaluation of the national surveillance system for point-prevalence of healthcare-associated infections in hospitals and in long-term care facilities for elderly in Norway, 2002-2008

**DOI:** 10.1186/1471-2458-11-923

**Published:** 2011-12-13

**Authors:** Agnes Hajdu, Hanne M Eriksen, Nina K Sorknes, Siri H Hauge, Hege L Loewer, Bjørn G Iversen, Preben Aavitsland

**Affiliations:** 1 Dept. of Hospital Epidemiology and Hygiene, National Center for Epidemiology, 1097, Gyáli út 2-6, Budapest, Hungary; 2 Dept. of Infectious Disease Epidemiology, Norwegian Institute of Public Health, 0403, P.O. Box 4404, Nydalen Oslo, Norway

## Abstract

**Background:**

Since 2002, the Norwegian Institute of Public Health has invited all hospitals and long-term care facilities for elderly (LTCFs) to participate in two annual point-prevalence surveys covering the most frequent types of healthcare-associated infections (HAIs). In a comprehensive evaluation we assessed how well the system operates to meet its objectives.

**Methods:**

Surveillance protocols and the national database were reviewed. Data managers at national level, infection control practitioners and ward personnel in hospitals as well as contact persons in LTCFs involved in prevalence data collection were surveyed.

**Results:**

The evaluation showed that the system was structurally simple, flexible and accepted by the key partners. On average 87% of hospitals and 32% of LTCFs participated in 2004-2008; high level of data completeness was achieved. The data collected described trends in the prevalence of reportable HAIs in Norway and informed policy makers. Local results were used in hospitals to implement targeted infection control measures and to argue for more resources to a greater extent than in LTCFs. Both the use of simplified Centers for Disease Control and Prevention (CDC) definitions and validity of data seemed problematic as compliance with the standard methodology were reportedly low.

**Conclusions:**

The surveillance system provides important information on selected HAIs in Norway. The system is overall functional and well-established in hospitals, however, requires active promotion in LTCFs. Validity of data needs to be controlled in the participating institutions before reporting to the national level.

## Background

With the ultimate goal being a reduction in the number of infections, national surveillance systems for healthcare-associated infections (HAI) typically aim to establish baseline rates over time, convince medical personnel to adopt preventive practices, evaluate control measures, and satisfy regulators [1].

In Norway (population 4.9 million), hospitals and long-term care facilities for elderly (LTCF) have legal obligations to implement HAI surveillance as part of the required infection control programme. While both types of institutions are obliged to have surveillance of HAI, only results from hospitals are demanded and requested by Free Hospital Choice Norway, a governmental initiative on patients' rights [2]. In 2008, there were 15,425 somatic beds in hospitals and 39,906 beds in institutions providing care for the elderly [3].

Previously, several national point-prevalence surveys of HAIs had been conducted with intervals of a few years [4,5]. To get comparable data which allows assessing trends over time, the Norwegian Institute of Public Health (NIPH) developed surveillance protocols both for hospitals and LTCFs. Since 2002, all facilities have been invited to participate in two national point-prevalence surveys each year. The overall prevalence of the four types of HAI included in the national surveillance was 5.1-6.4% in hospitals and 6.3-7.8% in LTCFs between 2002 and 2008.

In order to gain knowledge primarily about the system's performance in practice and, if necessary, improve its utility and efficiency, we conducted a comprehensive evaluation of the Norwegian surveillance system for point-prevalence of HAIs in hospitals and LTCFs.

## Methods

Guidelines for the evaluation of surveillance systems developed by the Centers for Disease Control and Prevention (CDC) and other key references were used in this assessment [6-8]. Attributes addressing primarily implementation and compliance issues were evaluated because of their importance to the national HAI surveillance.

### Description of the system

The evaluation focused on the surveillance system as a whole, starting with a description of the system and its components based on all available documents.

### Evaluation of system performance

**Figure 1:**
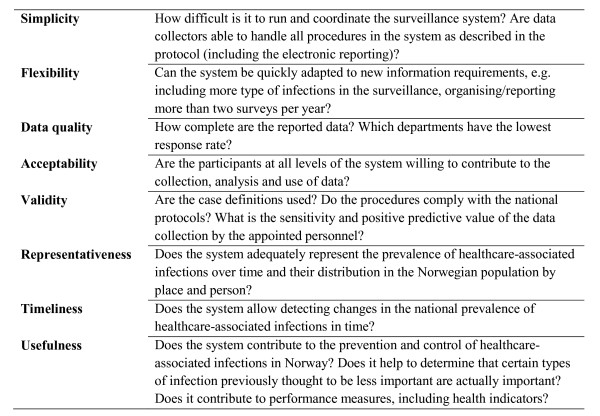
**Main focuses of the evaluation concerning the surveillance system's attributes**.

We assessed the system for simplicity, flexibility, data quality, acceptability, validity, representativeness, timeliness, and usefulness (Figure 1) taking into account the objectives of the surveillance: "*1) to measure baseline prevalence of infections, monitor trends and identify the distribution of HAIs in hospitals and in LTCFs; 2) to study further need for infection control (IC) measures and areas where incidence surveillance would be more adequate in hospitals; and 3) to increase the attention given to the prevention of HAIs and the importance of implementing IC programmes in LTCFs*" [9,10].

A combination of qualitative and quantitative methods was used to collect information: targeted surveys in hospitals and LTCFs, an on-site comparison of data reported by ward personnel to data reported by the evaluation team, open interviews with key personnel at the NIPH, and review of the database at national level.

In 2007, a structured electronic questionnaire (See Additional file 1: Survey among ICPs) was sent to one infection control practitioner (ICP) in each of the 50 main hospitals in Norway, and to one contact person per facility to 1065 LTCFs listed in a database of Norwegian health information. If no e-mail address for a LTCF was identified, the questionnaire's link was sent to the public e-mail address of the municipality with a request for forwarding it to the institution.

In addition, a one-page questionnaire (See Additional file 2: Survey among ward personnel) to assess compliance with the surveillance methodology was distributed to hospital ward personnel involved in prevalence data collection on the day of the national prevalence survey in May 2007. All departments in all hospitals were targeted.

A study of the validity of surveillance diagnoses by ward personnel was conducted in two municipal hospitals (referred to as hospital A and B in the text) in Southern and Eastern Norway in May 2007. Ward personnel collected and reported data on HAIs as part of the national prevalence survey. Independently, a team from NIPH also collected relevant data on the same patients in selected departments that previously had shown higher prevalence of HAI (general internal medicine, surgical and intensive care units). The evaluation team consequently used the surveillance case definitions as "gold standard" for case finding. Results of the two data collection methods were compared; sensitivity and positive predictive value of the data collection by ward personnel were calculated.

For practical reasons, review of detailed surveillance data at national level was limited to the surveillance period of 2004-2008. For human resources (e.g. work hours, number of persons involved), medians were calculated.

## Results

### Description of the system

**Figure 2:**
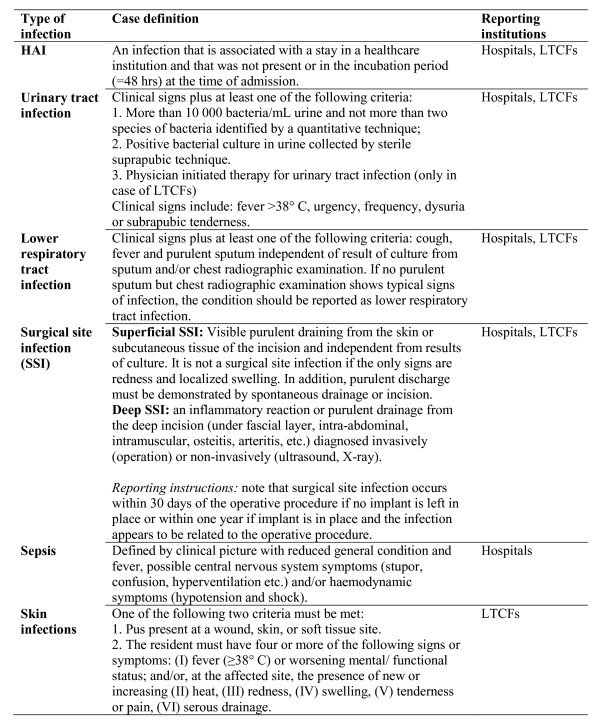
**Case definitions used (protocol version 2006)**. HAI, healthcare-associated infection; LTCF, long-term care facility. The case definitions of the national surveillance system for point-prevalence of healthcare-associated infections in hospitals and long-term care facilities for elderly in Norway are simplified and modified versions of the Centers for Disease Control and Prevention (CDC) definitions [31], except for skin infections where the definition of McGeer et al. is used [25].

The system is described in details both in the surveillance protocols that are available on NIPH's website http://www.fhi.no and in previous publications [9-11]. NIPH requires information on the occurrence of the following type of HAIs: infections of the urinary tract, lower respiratory tract and surgical site both in hospitals and LTCFs, whereas sepsis and skin infections only in hospitals and in LTCFs, respectively. The case definitions used and their references are shown in Figure 2. The following data are collected: name of institution, contact person, total number of patients on antimicrobial treatment, departmental response rate, non-participating departments; and by medical specialty/LTCF department: total number of in-patients/residents at 8 am on the survey day, total number of operated patients, total number of HAI by type of infection, total number of HAI acquired in the own institution, and total number of HAI acquired in another healthcare institution.

**Figure 3:**
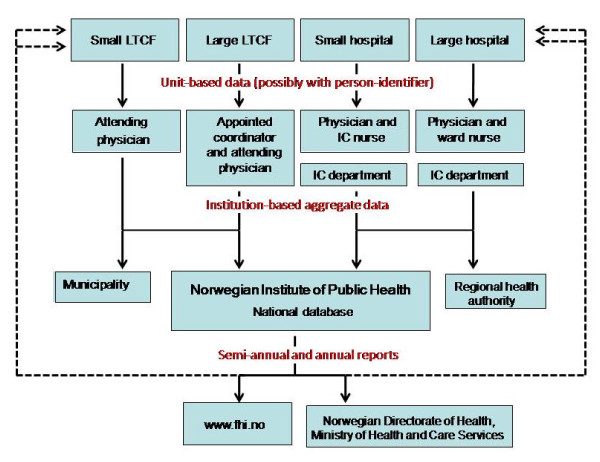
**Flow of information in the surveillance system**. LTCF, long-term care facility; IC, infection control. The figure shows the data flow in the national surveillance system for point-prevalence of healthcare-associated infections in hospitals and long-term care facilities for elderly in Norway.

E-mail reminders are sent to the institutions ahead of each survey, twice a year. Through the point-prevalence surveys, NIPH receives aggregate data on antimicrobial use by institution and on HAI by medical specialty/LTCF department. Figure 3 shows the flow chart of the system. A web tool that was implemented in 2004 allows registered users to submit data directly, compare their institution's data with aggregated national results, and create tables as well as figures by geographical distribution, medical specialty, hospital size and type of infections [11].

### Performance of the system

Response rate to the evaluation questionnaire was 58% (29/50) among ICPs in hospitals. All major hospitals provided answers. There were 435 respondents from 44 hospitals in the survey among ward personnel. The total number of healthcare workers who received the questionnaire is unknown.

Contact persons from 137 LTCFs answered (13%). All types of LTCFs by size of institution were equally represented among them.

Percentages presented below are based on the total number of responses to a question. Unless otherwise indicated, they include all answers (29 for hospitals, 137 for LTCFs).

### Simplicity

The surveillance system has a simple structure regarding the levels of data flow (Figure 3). Data collection forms require only the most necessary information for establishing the numerators and denominators. Users consider the protocols comprehensible at most points, though one in ten ICPs and contact persons noted that certain issues (aim of registration, unit-level form, and presentation of results) are not made completely clear (Tables 1 and 2). Eighteen of 28 (64%) ICPs and 82 of 127 (65%) contact persons in LTCFs and personnel at the national level find the reporting easy using the electronic surveillance tool.

**Table 1:** Hospital infection control practitioners' (n = 29) perception on the clarity of the national protocol

	**Very or quite clear **(%)	**Little unclear **(%)	**Very unclear **(%)	**Don't know **(%)
**Aim of the registration**	25 (86)	4 (14)	-	-

**What should be registered**	26 (90)	3 (10)	-	-

**Definition of HAI**	28 (97)	1 (3)	-	-

**Definitions of infections**	26 (90)	3 (10)	-	-

**Procedure of the survey**	26 (90)	3 (10)	-	-

**Unit-level form**	20 (69)	4 (14)	-	5 (17)

**Summary form**	25 (86)	1 (4)	-	3 (10)

**Reporting to NIPH**	23 (80)	3 (10)	1 (3)	2 (7)

**Presentation of results**	24 (83)	4 (14)	1 (3)	-

*HAI *healthcare-associated infection, *NIPH *Norwegian Institute of Public Health

**Table 2:** Long-term care facility contact persons' (n = 131) perception on the clarity of the national protocol

	**Very or quite clear **(%)	**Little unclear **(%)	**Very unclear **(%)	**Don't know **(%)
**Aim of the registration**	107 (82)	15 (11)	-	9 (7)

**What should be registered**	122 (93)	4 (3)	-	5 (4)

**Definition of HAI**	120 (92)	6 (4)	-	5 (4)

**Definitions of infections**	120 (92)	5 (4)	-	6 (4)

**Procedure of the survey**	123 (94)	3 (2)	-	5 (4)

**Unit-level form**	119 (91)	5 (4)	1 (1)	6 (4)

**Summary form**	118 (90)	5 (4)	-	8 (6)

**Reporting to NIPH**	112 (85)	8 (6)	2 (2)	9 (7)

**Presentation of results**	102 (78)	12 (9)	-	17 (13)

*HAI *healthcare-associated infection, *NIPH *Norwegian Institute of Public Health

### Flexibility

By allowing more prevalence surveys per year and additional types of infections to be included at the local level, the system shows its flexibility. Twenty-two of 28 (79%) hospitals organized 2-4 additional prevalence surveys besides the two national ones in 2006. Five (17%) hospitals survey both community- and hospital-acquired infections. Eight (28%) hospitals collect data on all types of HAI. In 13 (45%) hospitals, other variables (e.g. indwelling urinary catheter) are also registered. These additional data are not sent to the national level.

Eight of 125 (6%) LTCFs conduct additional prevalence surveys, 15 of 111 (14%) register more variables for local use (e.g. all infections requiring antibiotic therapy, cases with diarrhea, eye infection or infections caused by MRSA).

### Data quality

In the electronic database, data completeness is 100% for all variables, except the types of non-participating departments and number of patients on antibiotics on the day of the survey. For the latter variable, the total proportion of missing values was 30-51% in hospitals and 4-24% in LTCFs in the different surveys.

In 24 (83%) hospitals ICPs quality control the data before sending the results to NIPH (e.g. compare cases reported by ward personnel to clinical records and laboratory findings). Eighteen (62%) hospitals and 29 of 126 (23%) LTCFs reported to have some form of practical training provided to persons involved in data collection.

### Acceptability

Three (10%) hospitals and 44 of 134 (33%) LTCFs indicated non-participation in a previous prevalence survey. Reasons for non-participation listed by the three hospitals were technical problem, high workload due to incidence-based surveillance, and lack of resources. In LTCFs, with the number of answers shown in brackets, reasons included lack of resources or personnel (14), lack of surveillance protocol (10), no information on the timing (5), that it was forgotten by the personnel (5), or no request was received to conduct the survey (3). No knowledge on the implementation (1), technical problem (1) and heavy workload (1) were also mentioned.

Seventy-eight percent (73/94) of LTCFs with IC programme participated in the preceding prevalence survey, in contrast to the participation of 44% (16/36) of LTCFs without IC programme.

### Study of validity

In hospital A we included 48% (129/266) of patients and 43% (6/14) of departments, whereas in hospital B 26% (96/365) of patients and 25% (5/20) of departments were enrolled in the validation study. Merged results for identifying HAIs by the ward personnel in the two hospitals showed a sensitivity of 69% (9/13; 95% CI: 44-94%), specificity of 96% (203/212; 95% CI: 93-99%), positive predictive value of 50% (9/18; 95% CI: 27-73%), and negative predictive value of 98% (4/207, 95% CI: 96-100%), as compared to the independent assessment by the evaluation team. Detailed, case-to-case comparison of the findings revealed that among the nine HAIs registered both by ward personnel and the evaluation team, site of infection differed in four cases. In one hospital, seven community-acquired infections were reported by ward personnel as HAIs acquired in another healthcare institution.

### Other validity issues

Twenty-one (72%) hospitals organize both prevalence surveys on the dates announced by NIPH, eight (28%) organize one or both surveys on other dates. Nineteen (66%) hospitals provide denominator data as requested in the national protocol: patients present at the ward at 8 am on the day of the survey. Others include those patients only who had spent at least either 24 or 48 h in hospital before the survey.

Twenty-seven (93%) ICPs and 88 of 121 (73%) LTCF contact persons distribute the case definitions to the units before the prevalence survey.

Among the ward personnel that responded to the evaluation questionnaire, 348 of 431 (81%) received the case definitions during the prevalence survey in spring 2007. Compliance with the use of definitions was evaluated in this subgroup (Table 3). The majority of respondents had previous experience: 311 of 432 (72%) had been involved in three or more prevalence surveys, 55 (13%) in one or two.

**Table 3:** Compliance with the use of case definitions in national prevalence surveys reported by ward personnel in hospitals in spring 2007, Norway

	Always	Often	Sometimes	Rarely	Never	Total
	(%)	(%)	(%)	(%)	(%)	
Use of case definitions	134 (39)	68 (20)	91 (27)	39 (11)	9 (3)	n = 341

Use of 48 h cut-off*	107 (33)	67 (21)	47 (14)	57 (17)	49 (15)	n = 327

*Healthcare-associated infection: an infection that is associated with a stay in a healthcare institution and that was not present or in the incubation period (= 48 h) at the time of admission

Systematic, routine validation procedures have not been established at national level.

### Representativeness

In each of the prevalence surveys in the period of 2004-2008, between 79% and 96% of hospitals participated, and between 62% and 76% of somatic beds were covered. Approximately one third of LTCFs participated in the surveys between 2004 and 2008 (28-45% of all institutions with 36-52% of beds covered), except in the spring 2005 survey when the participation rate was 13%. Response rates of different medical specialties and LTCF departments are high, e.g. in spring 2007, 43 (86%) hospitals reported 100% response rate of medical specialties, and none had lower than 82%. Ninety-five percent (284/299) of LTCFs reported data from all departments.

### Timeliness

Unit-level forms are collected on the day of or, in large hospitals, within 1 or 2 days after the survey. Almost all participating institutions send their aggregated data to NIPH within 3 weeks of the survey. E-mail reminders are sent to hospitals only. In the web-based tool, users are able to see preliminary results once they have registered their data. Within approximately 2 months, the NIPH enters data sent by post or e-mail, checks data quality, clarifies errors, writes and publishes the semi-annual or annual report.

### Usefulness

**Figure 4:**
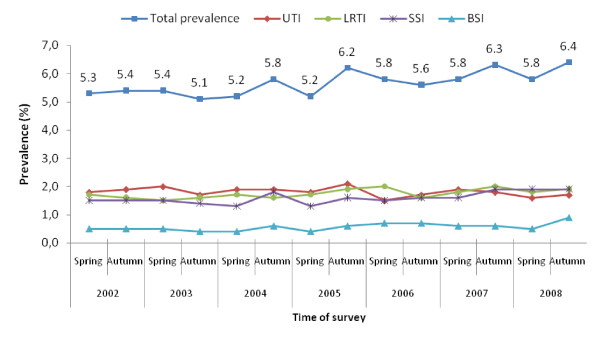
**Prevalence of healthcare-associated infections monitored in the national surveys in hospitals, Norway, 2002-2008**. UTI, urinary tract infection; LRTI, lower respiratory tract infection; SSI, surgical site infection; BSI, bloodstream infection.

**Figure 5:**
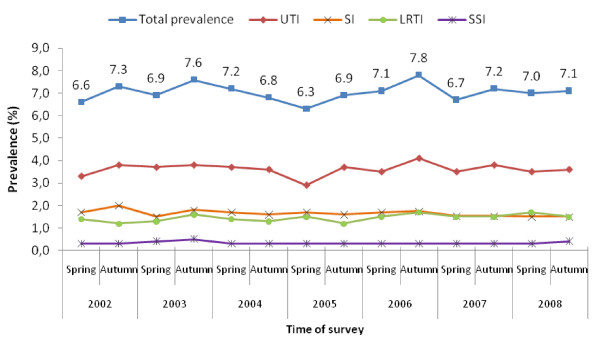
**Prevalence of healthcare-associated infections monitored in the national surveys in long term care facilities for elderly, Norway, 2002-2008**. UTI, urinary tract infection; SI, skin infection; LRTI, lower respiratory tract infection; SSI, surgical site infection.

National baselines for overall and infection-specific prevalence were established both in hospitals and LTCFs (Figures 4 and 5). In the frame of a governmental initiative, Free Hospital Choice Norway, launched in 2003, results of the surveys are made available to the public among several other indicators (e.g. waiting time) to help patients to get detailed information on the hospital in which they seek or undergo treatment. National prevalence data gave background information to a national action plan against HAIs issued by the Ministry of Health in 2004, and contributed to the formulation of a strategic goal of further improving surveillance of HAIs in Norway by means of incidence surveys [12]. The results also gave baseline data for a national hand hygiene campaign in hospitals and LTCFs in 2005 [13]. An ecologic study has shown that prevalence rates of HAI in a hospital may associate with the amount of hand hygiene products used [14].

Nine (31%) ICPs and four of 129 (3%) LTCF contacts used the prevalence results to argue for more resources for infection control at their institution. Nine (31%) hospitals and 21 of 121 (17%) LTCFs implemented local IC measures based on the results (e.g. revision of procedures, reduced use of permanent urinary catheters, education campaigns including hand hygiene).

### Human resources

At national level three persons are responsible for running the surveillance system: two advisors in NIPH and one external IT consultant. Maintenance and development of the web-based tool, including data quality assurance require annually 240 work hours of one advisor. Data entry and preparation of national reports require 80 work hours of two advisors; the web-based tool reduces this workload.

ICPs spend 6.8 work hours (range 2-40) on a prevalence survey, including preparation, data collection and supplementary work. Median workload is 2 h (range 1/4-25) for contact persons in LTCF, based on 105 answers.

## Discussion

Norway is among the few European countries which have implemented national surveillance of HAIs by periodic point-prevalence surveys. From 2011, the European Centre for Disease Prevention and Control (ECDC) will coordinate a joint prevalence survey in all member states [15]. Some of the strengths and challenges uncovered in our evaluation may be of particular interest to other countries which have or are planning to implement a similar surveillance system.

### Links to other surveillance systems on HAI

Another data source on HAI is the Norwegian surveillance system for healthcare-associated infections (NOIS). While the national coordination is organized similarly, the two systems are running independently from each other. The surveillance based on prevalence surveys is institution-wide and collects aggregate data on the presence of the most common type of HAIs on the survey day, whereas the NOIS is based on incidence surveys, collects patient-based data and at present, covers only surgical site infections after certain surgical procedures [16,17]. Results of the NOIS were therefore not utilized in this evaluation.

The prevalence of HAI in Norway has been comparable to findings in other countries [18-22], on the other hand several methodological differences may apply, including the selection of patients and hospitals, qualification and training of investigators and methods used to identify HAIs [23].

### Fulfillment of objectives

*Objective 1) *The surveillance system provides data regarding both hospitals and LTCFs, and has been reliably running since 2002. Each institution which has participated in at least a couple of surveys could set their own baseline values for prevalence and distribution of HAIs. National benchmarks have been established.

*Objective 2 and 3) *Several hospitals and LTCFs identified areas for improvement and initiated IC interventions based on their data. The national prevalence rates, increased awareness given to the area and known methodological shortcomings of the cross-sectional approach contributed to the development of targeted prospective surveillance (NOIS) in 2005, supported by a national action plan and related legal framework.

### Case definitions

In order to compare surveillance results with historical data, the same case definitions were used in the NIPH protocols as in previous prevalence surveys conducted in Norway. The use of simplified and modified CDC definitions instead of the comprehensive ones is controversial, and the question arises as to whether they are sufficiently valid and unambiguous for defining HAIs and whether they are accepted by those collecting the data [24]. These definitions were not validated before the implementation of the surveillance system. Another issue of concern is that the protocols for hospitals and for LTCFs contained the same set of definitions for lower respiratory tract infection and surgical site infection while the two facilities differ greatly in terms of professional resources and diagnostic capacity [25].

### Operation of the system

Regarding the flow of information, timeliness and data analyzed, the system seems very functional.

There was a certain variation seen in the data collection method and who was responsible for the registration locally. In national institution-wide surveillance it is challenging yet crucial to ensure similar understanding of the protocol and implementation of the same case finding method among all professionals involved [26]. In the evaluation, less than two third of ICPs in hospitals and less than one third of contact persons in LTCFs reported that they gave any form of practical training to the personnel involved in the registration.

The number of persons and time needed to run the surveillance was generally favorable. The web-based surveillance tool has proven to be successful both at local and national level.

### System attributes

The system is considered structurally simple and timely by those who are responsible for surveillance at national level.

The surveillance has very high coverage regarding hospitals. The proportion of participating LTCFs is less favorable perhaps because results from single institutions are not demanded and requested as opposed to hospitals in Free Hospital Choice Norway. In the survey with the lowest participation rate so far, no reminder letter was sent from national level to LTCFs in advance of the survey. Engagement in the surveillance might be improved in these institutions with more "direct marketing" considering the reported reasons for non-participation primarily being lack of information. Also, participation was considerably higher among those LTCF with IC programme than those without it, highlighting that availability of expertise, and presumably managerial support, may have an important impact on surveillance activities in this healthcare sector. Data on the number of patients/residents on antibiotic treatment was incomplete in the database both for hospitals and LTCFs; it might be due to scant medical documentation, more focus given to HAIs, or that NIPH had not prioritized this variable.

Validity of surveillance data is a challenge, and results of the evaluation also highlighted this problem. According to the ICPs, only two thirds of the hospitals used to provide the denominator data as required in the surveillance protocol. Additionally, reported compliance with the standard methodology scored low among ward personnel in hospitals, suggesting that prevalence data collection may be based on clinical judgment rather than the surveillance case definitions. This finding was supported by the results of the validity surveys, which indicated low sensitivity and positive predictive value of HAI registration by ward personnel compared to HAI ascertainment by the evaluation team. In approximately half of the hospitals ICPs routinely quality control the data collected before reporting their results to the national level which allows correction of the investigator bias and misunderstandings such as community-acquired infections being included. In other cases the error is most probably systematic given the ward personnel's reported experience in previous prevalence surveys and the relative stability of national rates over time. Repeated training and personal feedback on erroneous registration should be offered to those involved in the prevalence surveys. Additionally, validation is a key aspect to assure accuracy of HAI surveillance data [27].

The surveillance system has been proven to be useful. Though the overall prevalence has been relatively stable over the years, the results gave basis for actions at national level to facilitate prevention and control of particular types of HAIs. Further, more targeted interventions may be necessary to reach an actual reduction in prevalence rates [28]. At institutional level, results are more used in hospitals than in LTCFs to implement targeted IC measures, but also to argue for more IC resources. Even though few LTCFs reported action taken based on their results, the surveys undoubtedly increase awareness and knowledge on the issue of HAIs in care facilities and possibly foster the development of IC programmes in these institutions. Nonetheless, results of the prevalence surveys at institutional level should be interpreted carefully, even in case of repeated surveys, especially if events are rare. Concerning inter-hospital comparisons, the importance of adjustment for case-mix has been shown in previous studies [29,30]. The use of crude rates as quality indicators for hospitals should be avoided.

### Limitations of the evaluation

Response rate to the evaluation questionnaire was very low (13%) among contact persons in LTCFs. On the other hand, the proportion of respondents are neither unacceptable nor surprising if it is considered that approximately one-third of LTCFs is used to participate in any prevalence surveys, yet it hinders the generalization of the findings. The list of e-mail addresses are not fully up-to-date as approximately 10% of the e-mails returned with failure message.

A limited number of beds could be included in the surveys of validity due to feasibility reasons. Incomplete medical documentations sometimes made the identification of a HAI or conditions required by the case definitions difficult to the evaluation team, in these cases local ICPs were consulted to ensure the best decision. Nonetheless it is still possible that in these cases the surveillance diagnoses made were not always correct.

Due to lack of resources, the evaluation did not include the point of view of other users of the data, e.g. health politicians, hospital managers.

## Conclusions

The surveillance system for prevalence of HAIs in Norway has a proper public health rationale. Major strengths of the system are that it fulfills its main objectives and there seems to be a good balance between the system attributes and human resources needed to run the surveillance. The system is structurally simple, flexible, complete in reported data, useful in the outputs, and data are highly representative for hospitals. Nonetheless non-compliance issues with the use of standard methodology were shown and validity of data needs to be improved; this requires efforts both at national and local level. The surveillance system is well-established in hospitals, however, requires active promotion in LTCFs.

## Competing interests

The authors declare that they have no competing interests.

## Authors' contributions

AH drafted the evaluation protocol, performed the data analysis and drafted the manuscript, HME, BGI and PA participated in the design of the evaluation and critically revised the questionnaires, NKS, SHH and HLL were members of the evaluation team conducting the validity surveys. All authors revised and approved the final manuscript.

## Acknowledgements

We would like to thank all professionals that contributed to the evaluation by answering the questionnaires.

## Pre-publication history

The pre-publication history for this paper can be accessed here:

http://www.biomedcentral.com/1471-2458/11/923/prepub

## Supplementary Material

Additional file 1 **Survey among ICPs**. Questionnaire of the survey among infection control practitioners in hospitals [translation of the original Norwegian document]. Click here for file

Additional file 2 **Survey among ward personnel**. Questionnaire of the survey among hospital personnel involved in prevalence data collection in hospitals [translation of the original Norwegian document]. Click here for file
