# Supplementary material for: Evaluation of the national surveillance system for point-prevalence of healthcare-associated infections in hospitals and in long-term care facilities for elderly in Norway, 2002-2008
Source: BMC Public Health. 2011 Dec 13;11:923. doi: 10.1186/1471-2458-11-923 (PMC3265568; doi:10.1186/1471-2458-11-923)
Supplement: Additional file 1 — Survey among ICPs. Questionnaire of the survey among infection control practitioners in hospitals [translation of the original Norwegian document]. [file 1471-2458-11-923-S1.PDF]

Questionnaire of the survey among infection control practitioners in hospitals [translation of the original Norwegian document]

1. Name

2. Telephone number

3. E-mail

4. Position
   Infection control doctor
   Infection control nurse
   Other, specify: ________

5. Name of health authority

6. Name of hospital

7. County

8. Total number of somatic beds
   <100
   100-199
   200-399
   >400

9. Did your institution participate in the following national prevalence surveys?
   2002 Both in spring and autumn – only in spring – only in autumn – neither – don't know
   2003 **
   2004 **
   2005 **
   2006 **

   → *If no complete participation in 2006*
   10. Why did not your institution participate in both surveys in 2006? *multiple choice*
      Experienced technical problems
      Have not received information on the survey day
      Lack of personnel
      No resources to prioritise the surveys
      Saw no use of doing it
      Other surveillance method is used (eg. incidence)
      Don't know
      Other, spesify: ____________________

11. Did your institution participate in the prevalence survey in spring 2007?
   Yes
   No
   Don't know

   → *If no participation in spring 2007*
   12. Why did not your institution participate in spring 2007? *multiple choice*
      Experienced technical problems
      Have not received information on the survey day
      Lack of personnel
      No resources to prioritise the surveys
      Saw no use of doing it
      Other surveillance method is used (eg. incidence)
      Don't know
      Other, specify: ____________________

13. Have your institution organised prevalence surveys in addition to the two national ones in 2006?
    Yes
    No
    Don't know

    → *If Yes*
    14. How many prevalence surveys were organised <u>in addition to</u> the two national ones: ______

15. Do you conduct the prevalence surveys on the day of the national ones?
    Yes
    No
    Don't know
    Other, specify: ____________________

16. How do you get information on the national prevalence surveys? *multiple choice*
    MSIS-rapport (Bi-weekly communicable disease newsletter of NIPH)
    Via NIPH's website
    Via e-mail from NIPH
    Other, specify:___________________________

17. Do you think that the national prevalence protocol clear and simple regarding the

Aim of registration	– Very unclear – A little unclear – Quite clear – Very clear – Don't know
What should be registered	**
Definition of nosocomial infection	**
Definitions of the four nosocomial infections that should be registered	**
Procedure of the survey	**
The registration form to each ward	**
The summary registration form	**
Reporting the results to NIPH	**
Presentation of results	**

18. Are there any information you miss in the protocol or do you have other comments?:
____

19. Do you distribute the surveillance case definitions to the wards before/on the day of the survey?
    Yes, the definitions in the national protocol
    Yes, other definitions
    No
    Don't know
    Other, specify: ____________________

    → *If Yes*
    20. How do you distribute the definitions to the wards?
        Paper-based version together with the registration form
        Electronically (e.g. through intranet)
        Don't know
        Other, specify: ____________________

21. Do you think that the definitions in the national protocol are easy to understand?

Urinary tract infection	Yes – No – Don't know
Lower resp. tract infection	**
Superfic. surgical site inf.	**
Deep surgical site inf.	**
Sepsis	**

22. Do you think that the definitions correctly identify patients who have infection?

Urinary tract infection	Yes – No, too restrictive – No, too broad – Don't know
Lower resp. tract infection	**
Superfic. surgical site inf.	**
Deep surgical site inf.	**
Sepsis	**

23. Do you distribute the national registration form to the wards?
    - Yes, without alterations
    - Yes, with some alterations
    - No, we use our own form
    - Don't know

24. Which infections are registered during the prevalence surveys at your institution?
    - Only the four types of nosocomial infection that are required in the national protocol
    - All types of nosocomial infections
    - All infections (Both community- and hospital-acquired)
    - Don't know

    → *If only the four types required are registered*

    25. Do you want/ plan to register more types of infection?
        - Yes
        - No
        - Don't know

        → *If Yes*

        26. Which infections do you want/ plan to register in addition?:________________

27. Do you extend the registration to other variables for local use (eg. presence of urinary catheter)?
    - Yes
    - No
    - Don't know

28. Who collect the data during the prevalence surveys at your institution?
    - Only doctors
    - Only nurses
    - Nurse does the data collection and doctor approves the results
    - Infection control personnel
    - Don't know
    - Other, specify: ____________________

29. Which patients are included in the prevalence data you send to NIPH?
    - Total number of patients registered at the wards on the day of the survey
    - Only in-patients on the day of the survey
    - Only patients with at least 48 hours stay in the hospital
    - Don't know
    - Other, specify: ____________________

30. Do you check the quality of data before senting it to NIPH?
    - Yes
    - No
    - Don't know

    → *If Yes*

    31. What method do you use to quality check the prevalence data?: ________________________

32. Do you provide some form of training to the ward personnel involved in the data collection?
    - Yes
    - No
    - Don't know

33. Do you feed back results to the individual wards? *multiple choice*
    Yes, through intranet
    Yes, in written report
    Yes, in internal journal
    Yes, through personal contact
    No
    Don't know
    Other, specify: ____________________

34. Which reporting method did you use during the last national survey?
    Electronic surveillance tool on www.fhi.no
    Summary form (paper version) sent by post
    Summary form sent by e-mail
    Don't know
    Other, spesify: ____________________

35. Do you think that the reporting is easier with the electronic surveillance tool?
    Yes
    No
    Don't know

36. Have you experienced technical problems with the electronic surveillance tool?
    Yes
    No
    Don't remember

37. Have you used the new (version spring 2007) electronic reporting function in the surveillance tool?
    Yes
    No
    Don't know

38. Have you used the data of the national biannual reports published on www.fhi.no/ in the MSIS reports?
    Yes, for internal statistics, infection control report for the hospital
    Yes, for training
    Yes, for external, internal presentations
    No
    Don't know
    Other, specify: ____________________

39. Are you satisfied with the national biannual reports published on www.fhi.no/ in the MSIS reports?
    Yes
    No
    Don't know

40. What can be impoved regarding the presentation of the national results in these reports?:
____

41. Do you think that the results are representative for the nosocomial infection prevalence over time at your institution?
    Yes
    No, have lower prevalence
    No, have higher prevalence
    Don't know

42. Do you think that the prevalence surveys are useful part of infection control at your institution?
    Yes
    No
    Don't know

43. How much has the prevalence surveys helped to put focus on infection control at your institution?
    None
    Low degree
    Some degree
    High degree
    Don't know

44. Have you used the results of the prevalence surveys to argue for more resources for infection control?
    Yes
    No
    Don't know

45. Have the results of the prevalence surveys led to preventive or control measures at your institution?
    Yes
    No
    Don't know

    → *If Yes*
    46. Can you give examples of measures that were implemented?:
    ____

47. How many work hours do you use in total on a prevalence survey (including preparation, possibly implementation and complementary work)?: _____

48.How many persons are involved in theimplementation of prevalence surveys at your hospital (clinical and infection control personnel)?: ____

49. Other comments on the national prevalence surveillance system or on the prevalence surveys organised at your hospital: ____
