# Supplementary material for: Evaluation of the national surveillance system for point-prevalence of healthcare-associated infections in hospitals and in long-term care facilities for elderly in Norway, 2002-2008
Source: BMC Public Health. 2011 Dec 13;11:923. doi: 10.1186/1471-2458-11-923 (PMC3265568; doi:10.1186/1471-2458-11-923)
Supplement: Additional file 2 — Survey among ward personnel. Questionnaire of the survey among hospital personnel involved in prevalence data collection in hospitals [translation of the original Norwegian document]. [file 1471-2458-11-923-S2.PDF]

Evaluation of the national surveillance system for point-prevalence of healthcare-associated infections in Norway, 2002-2008

Questionnaire of the survey among hospital personnel involved in prevalence data collection in hospitals [translation of the original Norwegian document]

1. Name of hospital: ________________________________

2. Position:
   - Infection control doctor
   - Specialist (”overlege”)
   - Ward physician (”assistentlege”)
   - Resident doctor (”turnuslege”)
   - Infection control nurse
   - Chief nurse
   - Nurse
   - Other: _____________________________________

3. Have you been involved in prevalence data collection in the previous surveys? If yes, how many times?
   - Yes, number of surveys: 1 2 3 4 More than 4 times
   - No

4. Which infections are registered at your department?
   - All infections
   - Only nosocomial infections
   - Other:_________________

5. Have you received the definitions for nosocomial infections for the purpose of the prevalence survey?
   - Yes
   - No
   - Don’t know

6. How often do you use these definitions to define whether a patient has nosocomial infection?
   - Always
   - Often
   - Sometimes
   - Rarely
   - Never

7. How often do you use the 48 hour cut-off for hospital stay after admission to define whether the patient has nosocomial infection?
   - Always
   - Often
   - Sometimes
   - Rarely
   - Never

8. Do you think that the definitions for nosocomial infections are easy to understand?

Urinary tract infection	Yes	No	Don’t know
Lower resp. infection	Yes	No	Don’t know
Surgical site infection	Yes	No	Don’t know
Sepsis	Yes	No	Don’t know

If No, why:____

9. Do you think that the definitions correctly identify patients who have infection?

Urinary tract infection	Yes	No, it is too restrictive	No, it is too broad	Don’t know
Lower resp. infection	Yes	No, it is too restrictive	No, it is too broad	Don’t know
Surgical site infection	Yes	No, it is too restrictive	No, it is too broad	Don’t know
Sepsis	Yes	No, it is too restrictive	No, it is too broad	Don’t know

10. Overall how many patients were registered by you during the current prevalence survey? _____ patients

11. How much extra work does the prevalence data collection impose on you? _____ minutes or _____ hours
